# Defining a Core Genome Multilocus Sequence Typing Scheme for the Global Epidemiology of Vibrio parahaemolyticus

**DOI:** 10.1128/JCM.00227-17

**Published:** 2017-05-23

**Authors:** Narjol Gonzalez-Escalona, Keith A. Jolley, Elizabeth Reed, Jaime Martinez-Urtaza

**Affiliations:** a Center for Food Safety and Applied Nutrition, Food and Drug Administration, College Park, Maryland, USA; b Department of Zoology, University of Oxford, Oxford, United Kingdom; c The Milner Centre for Evolution, Department of Biology and Biochemistry, University of Bath, Bath, Somerset, United Kingdom; Mayo Clinic

**Keywords:** whole-genome sequencing (WGS), core genome multilocus sequence typing, cgMLST, Vibrio parahaemolyticus, clinical, phylogenetic analysis, phylogeny, single nucleotide polymorphism (SNP)

## Abstract

Vibrio parahaemolyticus is an important human foodborne pathogen whose transmission is associated with the consumption of contaminated seafood, with a growing number of infections reported over recent years worldwide. A multilocus sequence typing (MLST) database for V. parahaemolyticus was created in 2008, and a large number of clones have been identified, causing severe outbreaks worldwide (sequence type 3 [ST3]), recurrent outbreaks in certain regions (e.g., ST36), or spreading to other regions where they are nonendemic (e.g., ST88 or ST189). The current MLST scheme uses sequences of 7 genes to generate an ST, which results in a powerful tool for inferring the population structure of this pathogen, although with limited resolution, especially compared to pulsed-field gel electrophoresis (PFGE). The application of whole-genome sequencing (WGS) has become routine for trace back investigations, with core genome MLST (cgMLST) analysis as one of the most straightforward ways to explore complex genomic data in an epidemiological context. Therefore, there is a need to generate a new, portable, standardized, and more advanced system that provides higher resolution and discriminatory power among V. parahaemolyticus strains using WGS data. We sequenced 92 V. parahaemolyticus genomes and used the genome of strain RIMD 2210633 as a reference (with a total of 4,832 genes) to determine which genes were suitable for establishing a V. parahaemolyticus cgMLST scheme. This analysis resulted in the identification of 2,254 suitable core genes for use in the cgMLST scheme. To evaluate the performance of this scheme, we performed a cgMLST analysis of 92 newly sequenced genomes, plus an additional 142 strains with genomes available at NCBI. cgMLST analysis was able to distinguish related and unrelated strains, including those with the same ST, clearly showing its enhanced resolution over conventional MLST analysis. It also distinguished outbreak-related from non-outbreak-related strains within the same ST. The sequences obtained from this work were deposited and are available in the public database (http://pubmlst.org/vparahaemolyticus). The application of this cgMLST scheme to the characterization of V. parahaemolyticus strains provided by different laboratories from around the world will reveal the global picture of the epidemiology, spread, and evolution of this pathogen and will become a powerful tool for outbreak investigations, allowing for the unambiguous comparison of strains with global coverage.

## INTRODUCTION

Vibrio parahaemolyticus is an important human foodborne pathogen whose transmission is associated with the consumption of contaminated seafood (1). Most V. parahaemolyticus strains that are considered pathogenic carry genes encoding thermostable direct hemolysin (*tdh*) and/or thermostable direct hemolysin-related hemolysin (*trh*) (2). Usually, these potential pathogenic strains represent a small fraction of all environmental strains (3). In addition to these two virulence genes, pathogenic V. parahaemolyticus strains carry other virulence-related genes, usually located in pathogenicity islands (4–7).

The V. parahaemolyticus “pandemic clonal complex” has been the dominant clone causing diseases around the world (3, 8–14). The emergence and cross-border spreading of strains, mostly belonging to sequence type 3 (ST3), raised public health concerns regarding the possibility of a pandemic spread, an uncharacteristic trait for V. parahaemolyticus. It was believed that this pandemic strain was the only strain that was spreading among distant regions. However, recent findings have shown that this was not the case, and other V. parahaemolyticus strains belonging to diverse clonal complexes have been spreading between Asia and other parts of the world (15–18). The dispersal routes of these strains remain uncertain at the moment, but at least three different mechanisms have been identified as being associated with the introduction of pathogenic V. parahaemolyticus: ballast water, ocean currents, and transport of oysters or other mollusks between regions (11, 15, 16).

A first glance into the population structure and diversity of V. parahaemolyticus populations was accomplished by the establishment of the multilocus sequence typing (MLST) scheme for V. parahaemolyticus (19) and a centralized database (http://pubmlst.org/vparahaemolyticus) in 2008. This MLST database has enabled researchers from around the world to compare isolates. Currently, more than 2,477 strains from diverse regions of the world, belonging to 1,681 STs, are available for analyses. Genetic variants identified as prevalent in the different regions of the world can be mapped to identify potential connections between populations from diverse geographical areas and delineate potential routes of dispersion. Although useful, MLST is based on sequence analysis of 7 chosen housekeeping genes and therefore lacks enough resolution when used in outbreak scenarios to discriminate between related and unrelated strains at the ST level (19).

The prices for performing whole-genome sequencing (WGS) have decreased dramatically during the last 5 years, with genomes costing around $50 to $100 USD. Scientists have been using WGS to reanalyze historical collections of pathogens and outbreak strains, resulting in a new way of performing outbreak investigations. WGS analyses, such as WGS-single nucleotide polymorphism (WGS-SNP) (20–26) and core genome MLST analyses (15, 16, 27–33), have been used extensively for epidemiological trace back investigations of outbreaks. WGS data analyses allow us to better understand both population dynamics and the mechanisms which contribute to increased virulence among foodborne bacterial pathogens.

cgMLST schemes have already been successfully used for the analysis of different epidemiological investigations, such as the two recent V. parahaemolyticus outbreaks in Maryland (pandemic ST3 strains in MD in 2014 and a retrospective analysis of ST8 strains in MD in 2010) (15), the identification of a novel clone of V. parahaemolyticus causing infections in Peru (16), and the description of an emergent V. parahaemolyticus pathogenic strain (ST631) causing illnesses in the North Atlantic coast of the United States (34). All of the cgMLST schemes used in these analyses were custom-made for each strain type and according to a specific epidemiological context where strains were very similar and shared most of the genes with the reference strain (>83%) (12, 15, 16, 34). Therefore, there is a need to generate a portable, standardized, and more advanced system for the analysis of V. parahaemolyticus strains. Using WGS data will introduce a higher level of resolution and discrimination into the study of populations collected from all around the world, which can be analyzed using a universal cgMLST scheme for V. parahaemolyticus.

To establish this universal V. parahaemolyticus cgMLST scheme, we sequenced 92 V. parahaemolyticus genome representatives from the STs prevailing in different areas of the world. We used the genome of strain RIMD 2210633, which contained 4,832 total genes, as a reference, of which 2,254 genes were selected to create the new V. parahaemolyticus cgMLST scheme after analyzing those 92 genomes. Additionally, another 142 genomes available at NCBI were included in the study to evaluate the performance of the new cgMLST scheme. The cgMLST analysis was able to distinguish related and unrelated strains, including those with the same ST, clearly showing its enhanced resolution over conventional MLST analysis. The sequences obtained from this work were deposited and are available online in a public cgMLST V. parahaemolyticus database (http://pubmlst.org/vparahaemolyticus).

## RESULTS

### Sequencing of representative strains of V. parahaemolyticus for setting up the cgMLST scheme.

Ninety-two V. parahaemolyticus strains, previously used for setting up the MLST scheme for this bacterium (19), were sequenced to reach >25× average coverage using MiSeq (Illumina) (Table 1). Genome sequences with low coverage (<25×) usually result in low sequencing qualities and incorrect assemblies. Forty-eight additional strains previously sequenced by Ion Torrent (5) were resequenced by MiSeq in order to generate better-quality genomes (Table 2) and to validate the cgMLST scheme. *In silico* multilocus sequence typing (MLST; http://pubmlst.org/vparahaemolyticus) analysis of the *de novo* assembled contigs confirmed the identity of every V. parahaemolyticus strain (Tables 1 and 2).

**TABLE 1:** List of V. parahaemolyticus strains sequenced in this study

Isolate	CFSAN no.	Yr	Country	Source	ST^*a*^	Serotype	Accession no.	Coverage (×)
428/00	CFSAN018752	1998	Spain	C	17	O4:K11	LHAU00000000	145
30824	CFSAN018753	1999	Spain	C	17	O4:K11	LHAV00000000	88
9808/1	CFSAN018754	2004	Spain	C	3	O3:K6	LHAW00000000	131
UCM-V441	CFSAN018755	2002	Spain	E	52	O4:Kunk	LHAX00000000	107
UCM-V586	CFSAN018756	2003	Spain	E	45	O8:K22	LHAY00000000	114
906-97	CFSAN018757	1997	Peru	C	3	O3:K6	LHAZ00000000	127
357-99	CFSAN018758	1999	Peru	C	19	O3:Kunk	LHBA00000000	148
K0976	CFSAN001174	2004	USA	E	50	O6:K18	LHBB00000000	73
K1068	CFSAN018760	2004	USA	E	61	O5:Kunk	LHBC00000000	83
K1297	CFSAN018761	2004	USA	E	12	O5:K17	LHBD00000000	102
K1314	CFSAN018762	2004	USA	E	12	O4:K63	LHBE00000000	34
K1202	CFSAN018763	2004	USA	E	43	O4:K63	LHBF00000000	115
K1322	CFSAN018764	2004	USA	E	58	O3:K56	LHBG00000000	108
K1186	CFSAN018765	2004	USA	E	58	O3:K20	LHBH00000000	72
K1296	CFSAN018766	2004	USA	E	9	O10:K68	LHBI00000000	77
K1303	CFSAN018767	2004	USA	E	20	O1:Kunk	LHBJ00000000	131
NY3547	CFSAN001172	1998	USA	E	98	O4:K55	LHQW00000000	53
ATCC 17802	CFSAN022339	1951	Japan	C	1	O1:K1	MQUE00000000	92
K1193	CFSAN022890	2004	USA	E	15	O1:K9	SRR5070562	77
K1317	CFSAN022891	2004	USA	E	23	O1:K54	SRR5070560	129
K1302	CFSAN022892	2004	USA	E	50	O1:K25	SRR5070559	50
48262	CFSAN022893	1990	USA	C	43	O1:K56	SRR5070561	93
HC-01-22	CFSAN022894	2001	USA	C	43	O4:K63	SRR5070563	78
049-2A3	CFSAN022895	1997	USA	E	57	O4:K29	SRR5070568	65
HC-01-20	CFSAN022896	2001	USA	E	199	O1:Kunk	SRR5070567	96
M25-0B	CFSAN022897	1993	USA	E	22	O4:Kunk	SRR5070565	84
HC-01-06	CFSAN022898	2001	USA	E	199	O1:Kunk	SRR5070566	37
9546257	CFSAN022899	1995	USA	C	32	O4:K8	SRR5070569	144
98-506-B102	CFSAN022900	1998	USA	E	30	O5:K11	SRR5070574	91
98-506-B103	CFSAN022901	1998	USA	E	30	O5:K11	SRR5070571	112
98-513-F51	CFSAN022902	1998	USA	E	34	O4:K9	SRR5070570	95
98-548-D11	CFSAN023517	1998	USA	E	34	O4:K9	SRR5070572	110
98-605-A9	CFSAN023518	1998	USA	E	30	O5:K17	SRR5070573	43
98-605-A10	CFSAN023519	1998	USA	E	30	O5:K17	SRR5070586	99
99-524-A9	CFSAN023520	1999	USA	E	53	O3:K34	SRR5070584	98
99-780-C12	CFSAN023521	1999	USA	E	29	O11:Kunk	SRR5070588	148
DI-B11	CFSAN023522	1999	USA	E	54	O1:K22	SRR5070587	110
DI-A8	CFSAN023523	2000	USA	E	46	O1:K30	SRR5070585	136
DI-B-6-4	CFSAN023524	2000	USA	E	47	O1:K30	SRR5070601	102
CP-B-5	CFSAN023525	2000	USA	E	23	O1:K54	SRR5070598	132
DI-B-1	CFSAN023526	2000	USA	E	23	O1:K54	SRR5070600	82
DI-A-6-1	CFSAN023527	2000	USA	E	24	O1:K55	SRR5070597	142
DI-E5	CFSAN023528	2000	USA	E	60	O1:K55	SRR5070599	79
DI-B9	CFSAN023529	1999	USA	E	25	O1:K56	SRR5070649	103
DI-H8	CFSAN023530	1999	USA	E	26	O1:K56	SRR5070650	89
DI-C2	CFSAN023531	1999	USA	E	35	O4:K9	SRR5070648	70
DI-C5	CFSAN023532	1999	USA	E	35	O4:K9	SRR5070651	65
U5474	CFSAN023549	1980	Bangladesh	C	87	O3:K6	SRR5071102	93
PMA 1.5	CFSAN023550	2005	Chile	E	28	O3:K6	SRR5071104	24
PMA 2.5	CFSAN023551	2005	Chile	E	10	O4:Kunk	SRR5071129	30
PMA 3.5	CFSAN023552	2005	Chile	E	16	O4:Kunk	SRR5071130	71
PMA 16.5	CFSAN023553	2005	Chile	E	48	O4:K12	SRR5071131	95
PMA 45.5	CFSAN023555	2005	Chile	E	49	O3:K6	SRR5071133	122
PMA 79	CFSAN023557	2004	Chile	E	56	O2:Kunk	SRR5071135	43
PMA 112	CFSAN023558	2004	Chile	E	6	O3:K6	SRR5071136	45
PMA 189	CFSAN023559	2004	Chile	E	7	O3:K6	SRR5071134	136
PMA 337	CFSAN023560	2004	Chile	E	11	O7:Kunk	SRR5071137	59
PMA 339	CFSAN023561	2004	Chile	E	55	O4:Kunk	SRR5071139	36
PMA 3316	CFSAN023562	2004	Chile	E	13	O3:K6	SRR5071141	73
VpHY145	CFSAN023563	1999	Thailand	C	3	O4:K68	SRR5071143	83
KXV-641	CFSAN023564	1998	Japan	C	3	O1:K25	SRR5071140	52
AN-2189	CFSAN023565	1998	Bangladesh	C	3	O4:K68	SRR5071142	80
AP-11243	CFSAN023566	2000	Bangladesh	C	3	O1:Kunk	SRR5071144	59
PMA 109.5	CFSAN023556	2005	Chile	E	3	O3:K6	SRR5071138	33
PMA 37.5	CFSAN023554	2005	Chile	E	3	O3:K6	SRR5071132	37
TX2103	CFSAN023541	1998	USA	C	3	O3:K6	SRR5071094	103
BAC-98-3372	CFSAN023542	1998	USA	C	3	O3:K6	SRR5071092	104
BAC-98-3374	CFSAN023543	1998	USA	C	42	O3:K6	SRR5071095	118
BAC-98-4092	CFSAN023544	1998	USA	C	3	O3:K6	SRR5071096	126
AN-5034	CFSAN023545	1998	Bangladesh	C	3	O4:K68	SRR5071093	85
AO-24491	CFSAN023546	1999	Bangladesh	C	3	O1:K25	SRR5071106	94
VpHY191	CFSAN023547	1999	Thailand	C	3	O1:K25	SRR5071105	108
AN-16000	CFSAN023548	1998	Bangladesh	C	3	O1:Kunk	SRR5071103	90
Vp81	CFSAN023533	1996	India	C	3	O3:K6	SRR5070652	96
Vp155	CFSAN023535	1996	India	C	3	O3:K6	SRR5071101	132
Vp96	CFSAN023536	1996	India	C	3	O3:K6	SRR5071097	92
Vp208	CFSAN023537	1997	India	C	3	O3:K6	SRR5071099	123
AN-8373	CFSAN023538	1998	Bangladesh	C	3	O3:K6	SRR5071098	100
Vp2	CFSAN023540	1998	South Korea	C	3	O3:K6	SRR5071100	95
029-1(b)	CFSAN001611	1997	USA	E	36	O4:K12	JNTW02000000	104
48057	CFSAN001612	1990	USA	C	36	O4:K12	JNTX02000000	118
K1198	CFSAN001614	2004	USA	E	59	O4:K12	JNTY02000000	150
10292	CFSAN001617	1997	USA	C	50	O6:K18	JNTZ02000000	85
48291	CFSAN001618	1990	USA	C	36	O12:K12	JNUA02000000	99
F11-3A	CFSAN001619	1988	USA	E	36	O4:K12	JNUB02000000	113
NY-3483	CFSAN001620	1998	USA	C	36	O4:K12	JNUC02000000	72
K1203	CFSAN001173	2004	USA	E	59	O4:K12	JNUD02000000	47
98-513-F52	CFSAN001160	1998	USA	E	34	O4:K9	JNUE02000000	39
10290	CFSAN001613	1997	USA	C	37	O4:K12	JNUF02000000	51
JJ21-1C	CFSAN001615	1990	USA	E	38	O4:Kunk	LHPD00000000	64
W9OA	CFSAN001616	1982	USA	E	59	O4:K12	LHPE00000000	39
VP43-1A	CFSAN001621	1992	USA	E	36	O4:Kunk	LHQV00000000	92

a C, clinical; E, environmental.

**TABLE 2:** List of V. parahaemolyticus genomes from NCBI used for further testing of the newly created cgMLST

Isolate	CFSAN no.^*a*^	Yr	Country	Source^*b*^	ST	Serotype^*c*^	Accession no.	Reference or source
From our lab								
MDVP1^*d*^	CFSAN007429	2012	USA	C	631	unk	JNSM02000000	This study
MDVP8^*d*^	CFSAN007430	2012	USA	C	631	unk	JNSN02000000	This study
MDVP9^*d*^	CFSAN007431	2012	USA	C	631	unk	JNSO02000000	This study
MDVP31^*d*^	CFSAN007432	2013	USA	C	631	unk	JNSP02000000	This study
MDVP35^*d*^	CFSAN007433	2013	USA	C	631	unk	JNSQ02000000	This study
MDVP41^*d*^	CFSAN007434	2013	USA	C	631	unk	JNSR02000000	This study
MDVP44^*d*^	CFSAN007435	2013	USA	C	631	unk	JNSS02000000	This study
MDVP45^*d*^	CFSAN007436	2013	USA	C	631	unk	JNST02000000	This study
MDVP2^*d*^	CFSAN007437	2012	USA	C	651	unk	JNSU02000000	This study
MDVP3^*d*^	CFSAN007438	2012	USA	C	652	unk	JNSV02000000	This study
MDVP4^*d*^	CFSAN007439	2012	USA	C	653	unk	JNSW02000000	This study
MDVP34^*d*^	CFSAN007440	2013	USA	C	653	unk	JNSX02000000	This study
MDVP5^*d*^	CFSAN007441	2012	USA	C	113	unk	JNSY02000000	This study
MDVP7^*d*^	CFSAN007442	2012	USA	C	34	unk	JNSZ02000000	This study
MDVP11^*d*^	CFSAN007443	2012	USA	C	1116	unk	JNTA02000000	This study
MDVP6^*d*^	CFSAN007444	2012	USA	C	677	unk	JNTB02000000	This study
MDVP10^*d*^	CFSAN007445	2012	USA	C	43	unk	JNTC02000000	This study
MDVP13^*d*^	CFSAN007446	2012	USA	C	678	unk	JNTD02000000	This study
MDVP14^*d*^	CFSAN007447	2012	USA	C	162	unk	JNTE02000000	This study
MDVP15^*d*^	CFSAN007448	2012	USA	C	679	unk	JNTF02000000	This study
MDVP39^*d*^	CFSAN007455	2013	USA	E	896	unk	JNTL02000000	This study
090-96-70^*d*^	CFSAN001595	1996	Peru	C	189a	O4:K8	JFFP02000000	This study
VP16MD^*d*^	CFSAN007449	2012	USA	C	3	unk	JNTG02000000	This study
VP17MD^*d*^	CFSAN007450	2012	USA	C	3	unk	JNTH02000000	This study
VP18MD^*d*^	CFSAN007451	2012	USA	C	3	unk	JNTI02000000	This study
MDVP19^*d*^	CFSAN007452	2010	USA	C	8	unk	JNTJ02000000	15
MDVP20^*d*^	CFSAN007453	2010	USA	C	8	unk	JNTK02000000	15
MDVP22^*d*^	CFSAN007454	2010	USA	E	676	unk	JNUO02000000	15
MDVP25^*d*^	CFSAN007456	2010	USA	E	810	unk	JNUK02000000	15
MDVP26^*d*^	CFSAN007457	2010	USA	E	811	unk	JNUL02000000	15
MDVP27^*d*^	CFSAN007458	2010	USA	E	34	unk	JNUM02000000	15
MDVP28^*d*^	CFSAN007459	2010	USA	E	768	unk	JNUN02000000	15
MDVP21^*d*^	CFSAN012491	2010	USA	E	8	unk	JNUG02000000	15
MDVP23^*d*^	CFSAN012492	2010	USA	E	8	unk	JNUH02000000	15
MDVP24^*d*^	CFSAN012493	2010	USA	E	8	unk	JNUI02000000	15
MDVP29^*d*^	CFSAN012494	2010	USA	E	8	unk	JNUJ02000000	15
281-09^*d*^	CFSAN025052	2009	Peru	C	120	O3:K59	LKQB00000000	16
283-09^*d*^	CFSAN025053	2009	Peru	C	120	O3:K59	LKQA00000000	16
C220-09^*d*^	CFSAN025054	2009	Peru	C	120	O3:KUT	LKQC00000000	16
C224-09^*d*^	CFSAN025055	2009	Peru	C	120	O3:K59	LKQD00000000	16
C226-09^*d*^	CFSAN025056	2009	Peru	C	120	O3:K59	LKQE00000000	16
C244-09^*d*^	CFSAN025057	2009	Peru	C	120	O3:K59	LKQF00000000	16
C235^*d*^	CFSAN025058	2009	Peru	C	120	O3:K59	LKQG00000000	16
PIURA 17^*d*^	CFSAN025059	2009	Peru	C	120	O3:K59	LKQH00000000	16
C237^*d*^	CFSAN025060	2009	Peru	C	120	O3:K59	LKQI00000000	16
239-09^*d*^	CFSAN025061	2009	Peru	C	120	O3:K59	LKQJ00000000	16
241-09^*d*^	CFSAN025062	2009	Peru	C	120	O3:K59	LKQK00000000	16
245-09^*d*^	CFSAN025063	2009	Peru	C	120	O3:K59	LKQL00000000	16
CO1409^*d*^	CFSAN025064	2009	Peru	C	120	O3:K59	LKQM00000000	16
CO1609^*d*^	CFSAN025065	2009	Peru	C	120	O3:K59	LKQN00000000	16
285-09^*d*^	CFSAN025066	2009	Peru	C	120	O3:K59	LKQO00000000	16
287-09^*d*^	CFSAN025067	2009	Peru	C	120	O3:K59	LKQP00000000	16
379-09^*d*^	CFSAN025068	2009	Peru	C	120	O3:K59	LKQQ00000000	16
P306^*d*^	CFSAN029653	2009	Peru	E	120	O3:K59	LKQR00000000	16
Guillen 151 Peru^*d*^	CFSAN029654	2009	Peru	E	120	O3:K59	LKQS00000000	16
P310^*d*^	CFSAN029656	2009	Peru	E	120	O3:K59	LKQT00000000	16
From other labs								
10-4287^*d*^	NA	2003	Canada	C	50	O6:K18	JYJU00000000	Unpublished data^*i*^
BB22OP^*g*^	NA	1995	Bangladesh	E	88	O4:K8	NC_019955.1, NC_019971.1	51
CDC_K4557^*e*^	NA	2006	USA	C	799	O1:K53	NC_021822.1, NC_021848.1	52
FDA_R31^*e*^	NA	2007	USA	E	23	O1:Kunk	NC_021847.1, NC_021821.1	52
RIMD 2210633^*h*^	NA	2003	Japan	C	3	O3:K6	NC_004605.1, NC_004603.1	35
FORC_008^*d*^^,^^*e*^^,^^*g*^	NA	2004	South Korea	E	984	unk	NZ_CP009982.1, NZ_CP009983.1	Unpublished data^*j*^
UCM-V493^*d*^^,^^*e*^	NA	2002	Spain	E	471	O2:K28	CP007004, CP007005	53
CHN25^*g*^	NA	2011	China	E	395	unk	NZ_CP010884.1, NZ_CP010883.1	Unpublished data^*k*^
FORC_004^*e*^	NA	2014	South Korea	E	1628	unk	NZ_CP009848.1, NZ_CP009847.1	Unpublished data^*k*^
FORC_006^*d*^^,^^*e*^	NA	2014	South Korea	E	1630	unk	NZ_CP009765.1, NZ_CP009766.1	Unpublished data^*k*^
FORC_014^*e*^	NA	2015	South Korea	E	1629	unk	NZ_CP011407.1, NZ_CP011406.1	Unpublished data^*k*^
KVp10^*d*^	NA	2007	Sweden	E	1579	unk	MBTR01	Unpublished data^*l*^
R10B2_71^*d*^	NA	1997	USA	E	1556	unk	MCFR01	Unpublished data^*m*^
04-2192^*d*^	NA	2004	Canada	C	629	unk	LQCB01	Unpublished data^*n*^
04-2550^*d*^	NA	2004	Canada	C	630	unk	LRAH01	Unpublished data^*n*^
05-3133^*d*^	NA	2005	Canada	C	43	unk	LRAI01	Unpublished data^*n*^
05-4792^*d*^	NA	2005	Canada	C	199	unk	LPUZ01	Unpublished data^*n*^
07-2964^*d*^	NA	2007	Canada	C	8	unk	LRSV01	Unpublished data^*n*^
09-1772^*d*^	NA	2009	Canada	C	417	unk	LRSX01	Unpublished data^*n*^
09-3219^*d*^	NA	2009	Canada	C	36	unk	LRSW01	Unpublished data^*n*^
09-4436^*d*^	NA	2009	Canada	C	631	unk	LRAJ01	Unpublished data^*n*^
09-4661^*d*^	NA	2009	Canada	C	417	unk	LNTR01	Unpublished data^*n*^
09-4662^*d*^	NA	2009	Canada	C	417	unk	LRTH01	Unpublished data^*n*^
09-4665^*d*^	NA	2009	Canada	C	417	unk	LRFL01	Unpublished data^*n*^
09-4666^*d*^	NA	2009	Canada	C	417	unk	LQCC01	Unpublished data^*n*^
A0EZ383^*d*^	NA	2000	Canada	C	638	unk	LRSY01	Unpublished data^*n*^
A0EZ608^*d*^	NA	2000	Canada	C	36	unk	LRFM01	Unpublished data^*n*^
A0EZ664^*d*^	NA	2000	Canada	C	50	unk	LRFN01	Unpublished data^*n*^
A0EZ713^*d*^	NA	2000	Canada	C	50	unk	LRFO01	Unpublished data^*n*^
A1EZ679^*d*^	NA	2001	Canada	C	36	unk	LRSZ01	Unpublished data^*n*^
A1EZ919^*d*^	NA	2001	Canada	C	36	unk	LNTX01	Unpublished data^*n*^
A1EZ952^*d*^	NA	2001	Canada	C	43	unk	LRTI01	Unpublished data^*n*^
A2EZ523^*d*^	NA	2002	Canada	C	36	unk	LRTA01	Unpublished data^*n*^
A2EZ614^*d*^	NA	2002	Canada	C	43	unk	LRFP01	Unpublished data^*n*^
A2EZ715^*d*^	NA	2002	Canada	C	36	unk	LRFQ01	Unpublished data^*n*^
A2EZ743^*d*^	NA	2002	Canada	C	324	unk	LRFR01	Unpublished data^*n*^
A3EZ136^*d*^	NA	2003	Canada	C	3	unk	LRFS01	Unpublished data^*n*^
A3EZ634^*d*^	NA	2003	Canada	C	50	unk	LRTB01	Unpublished data^*n*^
A3EZ710^*d*^	NA	2003	Canada	C	43	unk	LRTC01	Unpublished data^*n*^
A3EZ711^*d*^	NA	2003	Canada	C	43	unk	LRTD01	Unpublished data^*n*^
A3EZ770^*d*^	NA	2003	Canada	C	50	unk	LRTE01	Unpublished data^*n*^
A3EZ799^*d*^	NA	2003	Canada	C	43	unk	LRTF01	Unpublished data^*n*^
A3EZ936^*d*^	NA	2003	Canada	C	1060	unk	LRTG01	Unpublished data^*n*^
A4EZ700^*d*^	NA	2004	Canada	C	43	unk	LOBT01	Unpublished data^*n*^
A4EZ703^*d*^	NA	2004	Canada	C	141	unk	LODO01	Unpublished data^*n*^
A4EZ724^*d*^	NA	2004	Canada	C	43	unk	LOHO01	Unpublished data^*n*^
A4EZ927^*d*^	NA	2004	Canada	C	3	unk	LOHN01	Unpublished data^*n*^
A4EZ964^*d*^	NA	2004	Canada	C	636	unk	LQGX01	Unpublished data^*n*^
A5Z1022^*d*^	NA	2005	Canada	C	15	unk	LRFT01	Unpublished data^*n*^
A5Z273^*d*^	NA	2005	Canada	C	?	unk	LQCD01	Unpublished data^*n*^
A5Z652^*d*^	NA	2005	Canada	C	36	unk	LQCE01	Unpublished data^*n*^
A5Z853^*d*^	NA	2005	Canada	C	3	unk	LQCF01	Unpublished data^*n*^
A5Z860^*d*^	NA	2005	Canada	C	43	unk	LQCS01	Unpublished data^*n*^
A5Z878^*d*^	NA	2005	Canada	C	36	unk	LQCT01	Unpublished data^*n*^
A5Z905^*d*^	NA	2005	Canada	C	36	unk	LQCU01	Unpublished data^*n*^
A5Z924^*d*^	NA	2005	Canada	C	36	unk	LQCV01	Unpublished data^*n*^
C140^*d*^	NA	2008	Canada	C	332	unk	LQCW01	Unpublished data^*n*^
C142^*d*^	NA	2008	Canada	C	417	unk	LPVA01	Unpublished data^*n*^
C143^*d*^	NA	2008	Canada	C	36	unk	LPVB01	Unpublished data^*n*^
C144^*d*^	NA	2008	Canada	C	36	unk	LPVC01	Unpublished data^*n*^
C145^*d*^	NA	2008	Canada	C	417	unk	LPVK01	Unpublished data^*n*^
C146^*d*^	NA	2008	Canada	C	1060	unk	LPVL01	Unpublished data^*n*^
C147^*d*^	NA	2008	Canada	C	36	unk	LPVM01	Unpublished data^*n*^
C148^*d*^	NA	2008	Canada	C	43	unk	LPVN01	Unpublished data^*n*^
C150^*d*^	NA	2008	Canada	C	417	unk	LPVU01	Unpublished data^*n*^
F1419^*d*^	NA	2006	Canada	C	43	unk	LRSU01	Unpublished data^*n*^
F30368^*d*^	NA	2006	Canada	C	8	unk	LRFV01	Unpublished data^*n*^
F4395^*d*^	NA	2006	Canada	C	36	unk	LRFU01	Unpublished data^*n*^
F63267^*d*^	NA	2006	Canada	C	3	unk	LRFW01	Unpublished data^*n*^
H11523^*d*^	NA	2006	Canada	C	36	unk	LRFY01	Unpublished data^*n*^
H18983^*d*^	NA	2006	Canada	C	36	unk	LRST01	Unpublished data^*n*^
H64024^*d*^	NA	2006	Canada	C	36	unk	LRFZ01	Unpublished data^*n*^
M59787^*d*^	NA	2006	Canada	C	36	unk	LRJZ01	Unpublished data^*n*^
T8994^*d*^	NA	2006	Canada	C	36	unk	LRGA01	Unpublished data^*n*^
W501^*d*^	NA	2006	Canada	C	635	unk	LRFX01	Unpublished data^*n*^
HS-06-05^*d*^	NA	2014	Canada	E	614	unk	LIRS01	Unpublished data^*n*^
ISF-29-3^*d*^	NA	2011	Canada	E	1518	unk	LFYM01	Unpublished data^*n*^
ISF-54-12^*d*^	NA	2011	Canada	E	1631	unk	LIRR01	Unpublished data^*n*^
S357-21^*d*^	NA	2010	Canada	E	102	unk	LFYN01	Unpublished data^*n*^
S372-5^*d*^	NA	2011	Canada	E	324	unk	LIRQ01	Unpublished data^*n*^
ISF-94-1^*d*^	NA	2011	Canada	E	1632	unk	LIRT01	Unpublished data^*n*^
RM-14-5^*d*^	NA	2014	Canada	E	1663	unk	LFXK01	Unpublished data^*n*^
Gxw_7004^*f*^	NA	2007	China	C	3	unk	LPZS01	Unpublished data^*o*^
Gxw_9143^*f*^	NA	2009	China	C	265	unk	LPZT01	Unpublished data^*p*^
K23^*d*^	NA	2013	India	E	1052	unk	LQGU01	54

a NA, not applicable.

b C, clinical; E, environmental.

c unk–unknown.

d MiSeq sequencing platform.

e PacBio sequencing platform.

f HiSeq sequencing platform.

g 454 sequencing platform.

h Sanger sequencing platform.

i J. Ronholm, N. Petronella, R. Kenwell, and S. Banerjee.

j J.-H. Lee, D.-H. Lee, S. Kim, H.-J. Ku, H. Y. Chung, H. Kim, S. Ryu, and S.-H. Choi.

k C. Zhu, B. Sun, T. Liu, H. Zheng, and L. Chen.

l J. W. Turner, R. N. Paranjpye, B. Collin, L. J. Pinnell, and J. Tallman.

m K. C. Liu.

n J. Ronholm, N. Petronella, and S. Banerjee.

o Y. Huang, H. Wang, Y. Pang, Z. Tang, Y. Zhou, and G. Sun.

p Y. Huang, H. Wang, Y. Pang, Z. Tang, Y. Zhou, C. Qu, L. Lan, C. Wei, and C. Wang.

### Development of a cgMLST for V. parahaemolyticus.

The initial setup of the cgMLST for V. parahaemolyticus using the genome of strain RIMD 2210633 as the reference genome (4,832 genes total) generated 3,709 potential core gene targets for use in the cgMLST scheme after eliminating duplicated, truncated, and accessory genes. RIMD 2210633 is a prototypic ST3 pandemic strain and was fully sequenced in 2003 using Sanger sequencing technology (35). Only core genes were used for constructing the cgMLST scheme. Of the 3,709 potential core genes identified in the comparison of strain RIMD 2210633 with seven other V. parahaemolyticus strains (BB22OP, CDC_K4557, FDA_R31, UCM-V493, FORC_008, FORC_006, and FORC_004), only 2,254 genes were present in every genome of the additional 92 V. parahaemolyticus strains used to define the final cgMLST scheme (see Table S1 in the supplemental material). These 92 strains represented a diverse set of strains isolated from environmental and clinical sources, as well as from different locations (Table 1).

### Implementation of the V. parahaemolyticus cgMLST website.

The cgMLST scheme was implemented into the BIGSdb database hosting the original MLST scheme for V. parahaemolyticus (http://pubmlst.org/vparahaemolyticus). This database allows for testing contigs of new V. parahaemolyticus genomes for the presence and typing of 2,254 genes. Briefly, the BIGSdb genome comparator tool performs a cgMLST analysis, which produces a color-coded cgMLST output (e.g., see Table S2), facilitating comparison among isolates (see Materials and Methods for specific details).

### Evaluation of the cgMLST target gene set.

**FIG 1:**
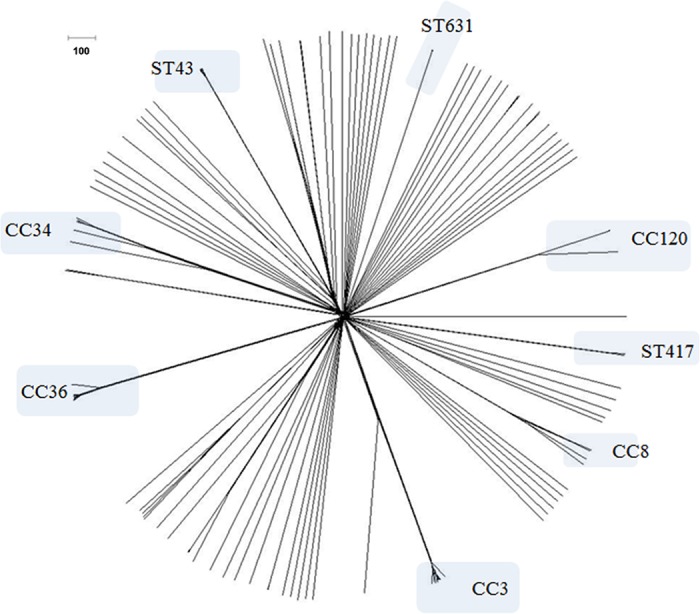
cgMLST analysis of the 234 V. parahaemolyticus genomes available at the V. parahaemolyticus MLST database using the genome comparator tool implemented within the MLST database (NeighborNet phylogenetic network). Visualization of the nexus file exported from the cgMLST analysis report in Splits Tree software (48). The names at the nodes were removed for easy visualization. The original tree with the nodes names is available in Fig. S1 in the supplemental material.

All V. parahaemolyticus genomes generated in this study, as well as a collection of 142 additional V. parahaemolyticus genomes available at NCBI (Table 2), were used to validate this cgMLST scheme (Fig. 1). The average percentage of cgMLST targets called was 99.21%. Only five assembled genomes contained incomplete loci: 97-10290 (two incomplete loci), Guillen_151_Peru (six incomplete loci), P310 (two incomplete loci), C148 (one incomplete locus), and HS-06-05 (seven incomplete loci). The output of this general analysis produced an informative Excel file (Table S2) composed of different sheets, with each one containing different results, as explained in Materials and Methods. cgMLST analysis for the 234 genomes available in the MLST database allowed a fast phylogenetic exploration of V. parahaemolyticus genomes (Fig. 1), clearly differentiating strains belonging to different STs, clustering strains with same STs, and allowing for further discrimination among strains within a specific ST.

### Evaluation of the cgMLST scheme using genomes of strains belonging to four known STs from outbreak-related and non-outbreak-related strains.

**FIG 2:**
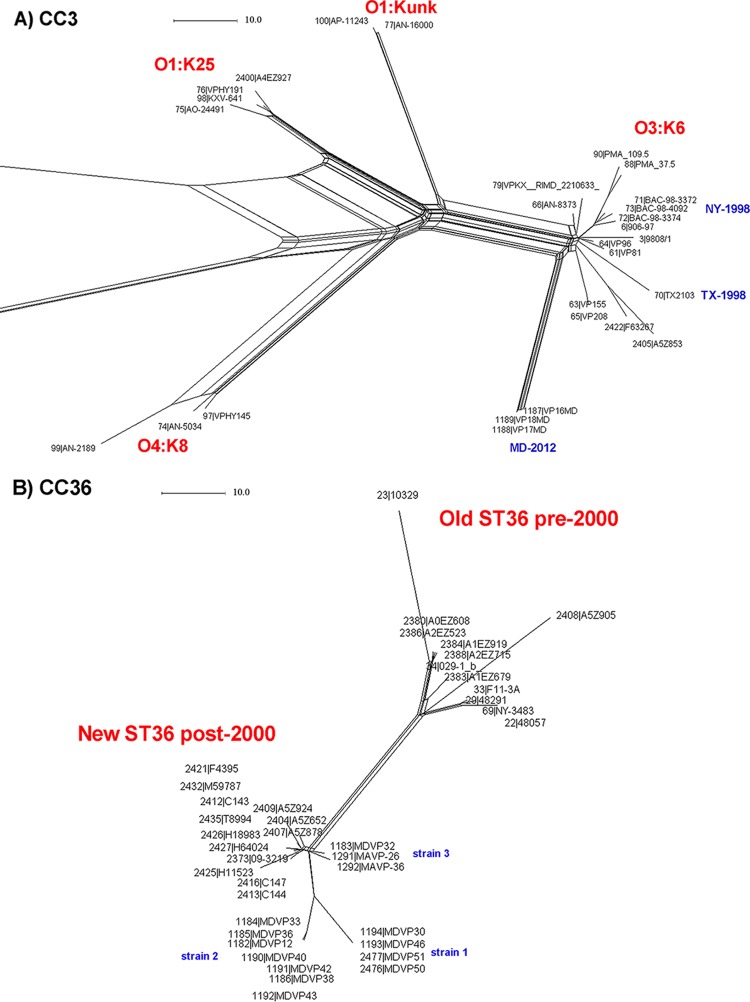

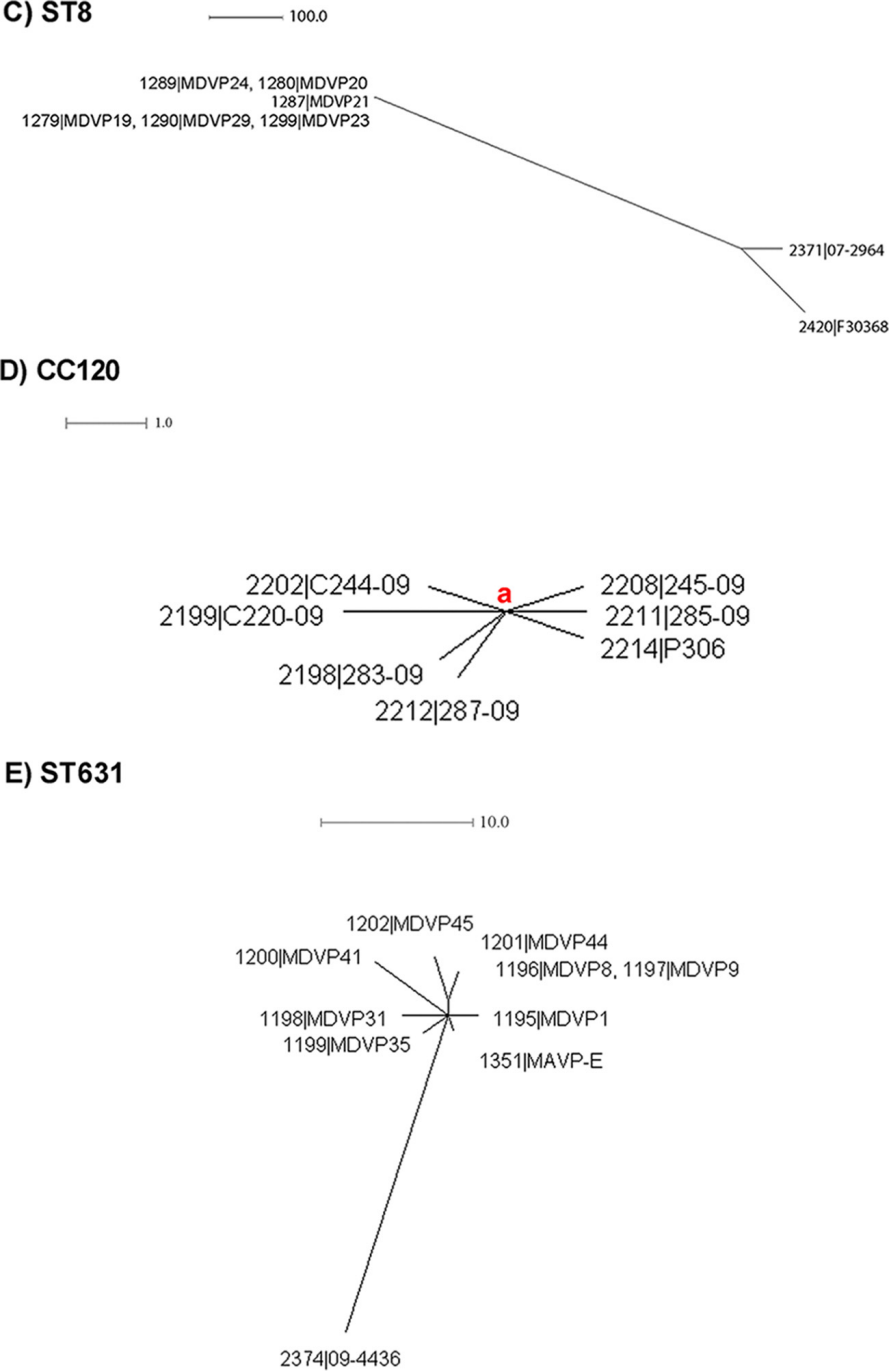
cgMLST analysis of representative V. parahaemolyticus strains from same outbreaks and/or non-outbreak related displaying the same ST identified in Fig. 1. (A) CC3 outbreak-related (12) and non-outbreak-related (19). (B) CC36-ST36 outbreak-related and non-outbreak-related strains (5, 19, 36). (C) CC8-ST8 outbreak-related and non-outbreak-related strains (15). (D) CC120 outbreak (Peru, 2009 [16]) strains 281-09, 241-09, 379-09, CO1409, CO1609, P310, Guillen_151_Peru, C226-09, C224-09, C235, PIURA_17, C237, and 239-09, were identical by cgMLST (represented by letter a). (E) ST631 strains (5, 34, 36). The scale represents the number of allele differences.

The performance of this cgMLST scheme was tested using six different sets of informative V. parahaemolyticus strains whose genomes were available and that clustered together in the global data set (Fig. 1). In addition to the unique pandemic clone of V. parahaemolyticus identified to date (clonal complex 3 [CC3]), other major groups with a relevance on a local or transnational scale were also analyzed: (i) strains belonging to ST36 (CC36) (outbreak related and non-outbreak related) (5, 19, 36) (Fig. 2B), (ii) strains belonging to ST8 (CC8) that were outbreak related, isolated in MD in 2010 (15) (Fig. 2C), (iii) strains belonging to ST120 (CC120) from the same outbreak (Peru, 2009) and that were recently characterized (16) (Fig. 2D), and (iv) strains belonging to ST631, a new emergent clone in the East Coast of the United States (5, 34, 36) (Fig. 2E).

### CC3.

The first test of the new cgMLST was performed using strains belonging to the pandemic clone CC3 using a panel of 30 strains (all ST3) epidemiologically unrelated, along with some additional strains collected in the course of a single epidemiological event (typically the same outbreak), including the recently reported strains MDVP16, MDVP7, and MDVP18 that caused a small outbreak in MD in 2014 (12) (Fig. 2A).

The cgMLST analysis of the genomes identified as CC3 by MLST consistently grouped the strains according to their serotype. Strains of serotypes O3:K6, O1:Kunk, O1:K25, and O4:K68 were efficiently discriminated and included in independent clusters. A high level of diversity was found within each cluster, even though these strains were highly related by PFGE profiling and random amplified polymorphic DNA (RAPD). The cgMLST was highly effective in separating strains that were less related to each other (e.g., see O3:K6 group). Noteworthy, cgMLST analysis showed that the first reported outbreaks of pandemic V. parahaemolyticus in the United States in 1998 (NY and TX) were caused by two different strains and differed by at least 14 loci from each other (detailed analysis can be found in Table S3). The strains causing the outbreak in MD in 2014 were grouped together and divergent from the original O3:K6 strains (old ST3 strains) by >30 loci. Strains MDVP17 and MDVP18 were undistinguishable and differed from MDVP16 by 1 locus, confirming that this outbreak in MD in 2014 was caused by a single strain.

### CC36.

CC36 includes strains typically causing infections in the Pacific Northwest United States and Canada (2, 19, 37). Figure 2B shows the analysis of strains belonging to CC36 from the United States and Canada isolated over the last 20 years from clinical and environmental sources. The cgMLST analysis clearly separated them into two distinct groups: strains isolated before 2000 (old or classic clone ST36) and strains isolated after 2000 (new clone ST36). The results of the cgMLST analysis can be found in Table S4. For illustration purposes here in this analysis, we focused on the known outbreak strains isolated in MD during the period of 2012 to 2013. In 2012, there was an outbreak on the East Coast of the United States caused by a unique ST36 clone (5, 38). This clone is represented by strain MDVP12 (grouped as strain 2 in the tree). However, as can be observed during the 2013 season, in the remaining MDVP strains, there were at least 3 different strains causing clinical cases during that year.

### CC8.

Strains belonging to this CC8 have been described as primarily causing illnesses in Asia (15); however, strains belonging to CC8 caused a small outbreak in MD in 2010 (15). Haendiges et al. (15) showed that these clinical ST8 strains were almost indistinguishable from strains isolated from oysters in MD and that they were different from other ST8 strains that were available at NCBI. Therefore, we chose these strains to test the performance of the newly developed V. parahaemolyticus cgMLST. Figure 2C shows the cgMLST analysis of these ST8 strains from an outbreak in MD in 2010 and their relationship to two other strains isolated in Canada. The qualities of the ST8 sequences available from NCBI were not “up to par” and were not included in this analysis, because they were sequenced at low coverage, and too many contigs were generated in their assembly (>300), indicative of the low quality of the sequences. The cgMLST analysis results (Table S5) clearly indicate that all ST8 strains from the MD outbreak in 2010 clustered together (differing up to 2 loci), revealing that the outbreak was caused by the same strain and differed by >500 loci from the ST8 strains isolated in Canada in 2006 and 2007.

### CC120.

Strains belonging to this CC120 and that were ST120 suddenly emerged in Peru during the course of a cross-country epidemic event in 2009 causing infections in different cities throughout the country (16). Figure 2D shows the cgMLST analysis with a set of 20 strains belonging to ST120 previously characterized by another cgMLST (custom reference based), causing an outbreak of gastroenteritis in Peru in 2009 (16). The results from the cgMLST analysis (Table S6) identified 11 of the 20 strains as undistinguishable, and the remaining 9 strains differed by 1 to 3 loci, indicating the high clonality of these strains and that they were indeed part of the same outbreak.

### ST631.

Strains belonging to ST631, which were also previously characterized by another custom-made cgMLST (34), were tested with the V. parahaemolyticus cgMLST. These strains belong to a new emergent V. parahaemolyticus clone causing the second highest number of V. parahaemolyticus illnesses in the East Coast of the United States. The cgMLST analysis results (Table S7) identified a highly clonal structure within this group (with two strains, MDVP8 and MDVP9, being undistinguishable) differing by between 1 and 10 loci, which contrasted with the differences found compared to ST631 strains isolated in Canada (>22 loci) (see Table S7).

## DISCUSSION

This study describes the implementation and evaluation of a cgMLST scheme for V. parahaemolyticus using a geographically diverse panel of V. parahaemolyticus strains with global coverage. A database from this study was created and is freely available online (http://pubmlst.org/vparahaemolyticus). The cgMLST scheme consisted of 2,254 target genes and was validated using 142 additional V. parahaemolyticus strains from diverse sources and geographical locations. The new database is a valuable and reliable tool for the unambiguous comparison of data generated from laboratories around the world.

The resequenced 140 genomes provided by this study to the NCBI database encompass a diverse repertoire of strains of historical importance. These genomes were instrumental in the creation of this universal cgMLST scheme for V. parahaemolyticus and represent a diverse set that can be used for other research endeavors, such as virulence typing, PCR detection of specific lineages, evolution, and spreading of different V. parahaemolyticus strains around the world (39, 40). This database will allow for testing contigs of new V. parahaemolyticus genomes for the presence and typing of 2,254 genes. The steady incorporation of new genomes into this database will improve surveillance of this important foodborne pathogen worldwide and provide early detection of new variants being introduced into locations where they are not usually found, as was shown for ST189 (17), ST3 in MD 2014 (12), ST8 in MD 2010 (15), and ST120 in Peru 2009 (16), among others.

The suggested analysis starts with running a default cgMLST analysis with all of the V. parahaemolyticus genomes available in the database, and the new V. parahaemolyticus genomes being tested can be localized in the NeighborNet tree (Fig. 1). This type of analysis allows for a fast phylogenetic examination of V. parahaemolyticus genomes. Then, a more detailed analysis can be produced that includes only the relevant strains contained in the initial tree that clustered with the V. parahaemolyticus genomes tested (Fig. 2). Also, two types of output of the analysis can be performed: a fast analysis output, in which only the allelic information is used, and a more detailed (although slower) output, where not only are the alleles differences, but also an alignment containing the sequences for all the variable genes (loci), are provided. The more detailed output (which is generated in order to be able to generate phylogenetic trees outside the website) can be used to perform additional tests, such as SNP-based phylogeny reconstruction using sequenced-based algorithms, such as maximum likelihood (41), time of evolution (42), or to find a specific sequence signature for an specific lineage or clone.

The evaluation of this universal V. parahaemolyticus cgMLST was performed using five sets of strains known to be part of the same outbreak or unrelated but having the same ST. As expected, cgMLST was extremely efficient in partitioning even among the highly clonal ST3 (pandemic strains), dividing the strains causing an outbreak in the United States in 1998 in two different locations (NY and TX) into two different groups (Fig. 2A). This result is in line with findings from other ongoing studies also identifying these two strains (TX and NY, 1998) having a different origin (our unpublished data). Furthermore, it partitioned the pool of strains in concordance with their serotypes, with all the O3:K6 strains clustering loosely together, while strains from each other serotype were grouped consistently according to serotype. This analysis also showed that the ST3 strains from the outbreak in MD in 2014 (12) were almost identical strains (only 1 SNP difference in one strain among the 2,254 genes analyzed) and very different from the other ST3 strains analyzed. This conclusion was not possible to arrive at previously due to the inherent problems with the sequence quality and analysis performed in the earlier publication (12).

A similar result was achieved with the other sets of strains employed for each individual analysis. Strains belonging to CC36 from the United States and Canada were separated by the V. parahaemolyticus cgMLST analysis into two distinct groups, as observed preliminarily elsewhere (5, 36), with strains isolated before 2000 (classic ST36 clone) and after 2000 (new ST36 clone). It also showed that ST36 strains causing an outbreak in 2013 in MD belonged at least to 3 different lineages. This example clearly shows the performance of the cgMLST for fast clustering and differentiation of strains during an outbreak. The overall MLST discriminatory power expressed by the formula of Simpson's index of diversity (D) for the genomes analyzed was 0.947, which shows that MLST is quite discriminatory but is not enough to discriminate within strains of the same ST. Overall, however, the D of cgMLST was 0.9921, showing a significantly higher discriminatory power than MLST.

This cgMLST analysis has several advantages compared to SNP-based methodologies: it is rapid, reproducible, there is no need for high-performance computers or bioinformatic skills, it allows easy visualization and location on the genome of the loci that differ between or among strains analyzed, the results can be easily transferred between different laboratories, and the information for each genome from all around the world will be stored in the database for future use. In contrast, a limitation of the cgMLST approach is that the analysis is reduced to only coding regions. Of the 4,832 open reading frames (ORFs) used as references (present in RIMD strain), only 50% are shared by the highly diverse V. parahaemolyticus strains used in this study, representing only a fraction of the genome. Therefore, if more detailed or enhanced resolution is needed, whole-genome MLST (wgMLST) using an uploaded annotated reference of a related strain (supported within the website) or a genome-wide SNP analysis is recommended.

V. parahaemolyticus is a natural inhabitant of a wide range of marine habitats, with a life cycle encompassing different stages as free-living organism in seawater, as a component of the microbiota of a vast range of marine organisms, but also as a pathogen in the human gut (43). As a result of this complex lifestyle, this organism is extremely diverse in terms of genomic variation, with a large genomic repertory which enables it to adapt and survive in different habitats under the constant variations in the environmental conditions typical of coastal areas. In addition to mutation, homologous recombination and horizontal gene transfer have been found to represent major contributions to genomic variation in V. parahaemolyticus populations in the need for a rapid adaptation to new habitats under changing environmental conditions (17, 19, 44, 45). These particular features make the phylogenetic analysis of V. parahaemolyticus especially challenging where the identification of the different sources contributing to genetic variation of genomes is needed. For all these reasons, the cgMLST scheme described here represents a notable advance in the genomic analysis of complex organisms, such as V. parahaemolyticus, providing a permanent platform to store available genomes, streamlining the analytical process with the selection of the core genes shared by all the genome and a rapid identification of the variation within each gene, without the need to deal with complex and time-consuming bioinformatics tools, and enabling an urgent response within a context of epidemiological investigation.

In conclusion, we have created a standardized cgMLST scheme that allows for fast typing of V. parahaemolyticus from WGS data in a publicly available database. This cgMLST scheme was tested with a diverse set of strains belonging to the same or unrelated outbreaks and was able to differentiate them accordingly, therefore showing a great potential for use in outbreak investigations. Application of this cgMLST scheme to V. parahaemolyticus strains collected by different laboratories around the world will help define the global picture of the epidemiology, spread, and evolution of this pathogen. All of this information will be critical in its application to outbreak investigations, providing a unique repository of genomes that can be used for unambiguous comparisons of data generated worldwide. Finally, since V. parahaemolyticus is a bacterium highly intertwined with environmental changes, it is our goal to develop a tool that would be able to integrate the results obtained from the cgMLST scheme analysis of the entire database, as it continues to grow, into a geographical visualization that together with environmental variables (e.g., salinity and temperature) would help to determine worldwide dispersal rates of this pathogen and help in modifying risk assessments for this bacterium in different regions.

## MATERIALS AND METHODS

### Bacterial strains and media.

The V. parahaemolyticus strains sequenced in this study are listed, along with their assigned CFSAN numbers, in Table 1. Strains were selected based on their origin, ST, and date of isolation, with representatives of all the major clinical clones of V. parahaemolyticus prevailing in the different regions of the world. All isolates were retrieved from storage (−80°C freezer), transferred to Luria-Bertani (LB) medium with 3% NaCl, and incubated at 37°C with shaking at 250 rpm. Strains were confirmed in the original studies as belonging to V. parahaemolyticus and subsequently confirmed in this study by *in silico* MLST and *in silico* presence of a V. parahaemolyticus-specific gene (Vp-toxR-AB029907) in the genome.

### DNA extraction and quantification.

Genomic DNA from each strain was isolated from overnight cultures using the DNeasy blood and tissue kit (Qiagen, Valencia, CA). The concentration was determined using a Qubit double-stranded DNA high-sensitivity (HS) assay kit and a Qubit 2.0 fluorometer (Thermo Scientific, Waltham, MA), according to each manufacturer's instructions.

### Whole-genome sequencing, contig assembly, and annotation.

Strains were sequenced (Table 1 and some in Table 2) using an Illumina MiSeq sequencer (Illumina, CA) with 2 × 250-bp paired-end chemistry, according to the manufacturer's instructions, with >25× average coverage. The genome libraries were constructed using the Nextera XT DNA sample prep kit (Illumina). Genomic sequence contigs were *de novo* assembled using default settings within CLC Genomics Workbench version 8.5.1 (Qiagen), with a minimum contig size threshold of 500 bp in length. The draft genomes were annotated using the NCBI Prokaryotic Genome Annotation Pipeline (PGAP [http://www.ncbi.nlm.nih.gov/genomes/static/Pipeline.html]) (46).

### *In silico* MLST phylogenetic analysis.

The initial analysis and identification of the strains were performed using an *in silico* V. parahaemolyticus MLST, based on information available at the V. parahaemolyticus MLST website (http://pubmlst.org/vparahaemolyticus/) and using Ridom SeqSphere+ software version 3.1.0 (Ridom, Münster, Germany). Seven loci (*dnaE*, *gyrB*, *recA*, *dtdS*, *pntA*, *pyrC*, and *tnaA*), previously described for V. parahaemolyticus (19), were used for MLST analysis. The same V. parahaemolyticus MLST database was also used to assign numbers for alleles and sequence types (STs).

### cgMLST target gene definition.

The cgMLST scheme for V. parahaemolyticus was created using Ridom SeqSphere software version 3.1.0, with the genome of strain RIMD 2210633 as a reference (Ridom, Münster, Germany). The cgMLST scheme was composed using the cgMLST target definer tool, using the default settings within the software. The reference genome contains 4,832 genes in total (35). The only seven closed V. parahaemolyticus genomes available at NCBI were used to establish a list of core and accessory genome genes (strains BB22OP, CDC_K4557, FDA_R31, UCM-V493, FORC_008, FORC_006, and FORC_004). Core genes, genes shared by all the strains queried, and accessory genes that were only present in some, but not all, of the queried genomes were identified. Genes that were present in more than one copy in any of the eight genomes were removed from the analysis. A genome-wide gene-by-gene cgMLST comparison was performed with every genome queried against the reference.

### Establishment of the cgMLST for V. parahaemolyticus website.

The V. parahaemolyticus MLST website (http://pubmlst.org/vparahaemolyticus/) is run using the BIGSdb platform (47) designed for gene-by-gene analysis of whole-genome assemblies. Establishing the cgMLST scheme was a matter of defining the core gene loci within the database and grouping these into a scheme. The first allele (allele 1) for each locus was defined from the RIMD 2210633 strain and added to the database in order to seed it. New variants of each locus were found using the BIGSdb manual Web-based scan tools and automated offline allele definer. This identified new variants by performing a BLAST query of the genome assembly against a database of known alleles. New alleles were assigned automatically if they had an identity of ≥98% with an existing allele over an alignment length of ≥98% of the allele and contained an initial start codon, a final stop codon, and were in frame with no internal stop codons. New alleles that did not match the description above were manually curated. Allele designations and positions for each locus in each genome assembly were recorded within the database.

### Genealogical reconstructions using the cgMLST scheme.

Gene-by-gene analysis was performed using the BIGSdb Genome Comparator tool (47). This analysis produced an output showing allelic variation at each locus, further categorized into loci that are (i) varied among all strains, (ii) same among all strains, and (iii) incomplete in some isolates; also included in the output are (iv) unique strains, (v) a distance matrix, and (vi) the parameters used for comparison. The distance matrix generated by the analysis is based on allelic differences across the cgMLST loci, with every locus with a different allele counted as a single difference in pairwise comparisons of isolates. The genealogies were reconstructed from this distance matrix using the NeighborNet algorithm (48) implemented in SplitsTree4 (49) and were either integrated into the PubMLST website or the desktop package was used.

### Evaluation of the cgMLST target gene set.

A collection of 142 additional V. parahaemolyticus genomes available at NCBI (Table 2) was used to validate the cgMLST scheme. Some of these genomes were sequenced *de novo*, because cgMLST performed best with high-quality sequences, which were those with >25× coverage and without indels due to homopolymers or sequencing errors that might arise from some sequencing techniques, such as 454 and Ion Torrent (Table 2). These strains have been isolated from various sources (environmental and clinical) around the world and constitute a diverse set of V. parahaemolyticus strains. Some of them belonged to the same outbreak, and others belonged to the same ST but were not epidemiologically related. All isolates have been previously evaluated by MLST (http://pubmlst.org/vparahaemolyticus). The index of discrimination or discriminatory power (D) of cgMLST and MLST was calculated using the Simpson's index of diversity, as described previously (50).

### Accession number(s).

The draft genome sequences for all 129 V. parahaemolyticus strains used in our analyses are available in GenBank under the accession numbers listed in Tables 1 (92 strains) and 2 (37 strains).

## Supplementary Material

Supplemental material
