# Supplementary material for: Defining a Core Genome Multilocus Sequence Typing Scheme for the Global Epidemiology of Vibrio parahaemolyticus
Source: J Clin Microbiol. 2017 May 23;55(6):1682–97. doi: 10.1128/JCM.00227-17 (PMC5442524; doi:10.1128/JCM.00227-17)
Supplement: Supplemental material [file supp_55_6_1682__index.html]

Defining a Core Genome Multilocus Sequence Typing Scheme for the Global Epidemiology of Vibrio parahaemolyticus — Supplemental material 

# Defining a Core Genome Multilocus Sequence Typing Scheme for the Global Epidemiology of Vibrio parahaemolyticus

## Supplemental material

- Supplemental file 1 -

  Table S1 (List of genes used for the cgMLST and sequence in strain RIMD 2210633)

  XLSX, 861K
- Supplemental file 2 -

  Table S2 (cgMLST analysis for the 234 *V. parahaemolyticus* genomes used in this study)

  XLSX, 5.5M
- Supplemental file 3 -

  Table S3 (cgMLST analysis of CC3 strains)

  XLSX, 494K
- Supplemental file 4 -

  Table S4 (cgMLST analysis of CC36 strains)

  XLSX, 565K
- Supplemental file 5 -

  Table S5 (cgMLST analysis of ST8 strains)

  XLSX, 190K
- Supplemental file 6 -

  Table S6 (cgMLST analysis of ST120 strains)

  XLSX, 321K
- Supplemental file 7 -

  Table S7 (cgMLST analysis of ST631 strains)

  XLSX, 204K
